# Proteome dataset of *Candida albicans* (ATCC10231) opaque cell

**DOI:** 10.1186/s13104-023-06661-z

**Published:** 2024-01-02

**Authors:** Gajanan Zore, Mazen Abdulghani, Santosh Kodgire, Rubina Kazi, Amruta Shelar, Rajendra Patil

**Affiliations:** 1 Dept. of Biotechnology, School of Life Sciences, Central University of Rajasthan, Ajmer, Bandersindri NH 8, Kishangarh Dist Ajmer (Rajasthan), India; 2 School of Life Sciences, Swami Ramanand Teerth Marathwada University, Nanded, 431606 MS India; 3 Division of Biochemical Sciences, CSIR-NCL, Pune-8, Pune, MS India; 4 Department of Biotechnology, Savitribai Phule Pune University, Ganeshkhind, Pune, 411007 MS India

**Keywords:** *Candida albicans*, Opaque, LC-MS/MS, Proteomics, Phenotypic switching, Mating type

## Abstract

**Objectives:**

*Candida albicans*, a polymorphic yeast, is one of the most common, opportunistic fungal pathogens of humans. Among the different morphological forms, opaque form is one of the least-studied ones. This opaque phenotype is essential for mating and is also reported to be involved in colonizing the gastrointestinal tract. Considering the significance of the clinical and sexual reproduction of *C. albicans*, we have investigated the morphophysiological modulations in opaque form using a proteomic approach.

**Data description:**

In the current investigation, we have used Micro-Liquid Chromatography-Mass Spectrometry (LC-MS/MS) analysis to create a protein profile for opaque-specific proteins. Whole-cell proteins from *C. albicans* (ATCC10231) cells that had been cultured for seven days on synthetic complete dextrose (SCD) medium in both as an opaque (test) and as a white (control) form cells were extracted, digested, and identified using LC-MS/MS. This information is meant to serve the scientific community and represents the proteome profile (SWATH Spectral Libraries) of *C. albicans* opaque form.

## Objective

*Candida albicans* is a polymorphic, opportunistic pathogen of humans that exists in various morphological forms and sizes, including yeast, hyphae, pseudohyphae, chlamydospores, and white and opaque cells and in two different forms of growth, i.e., planktonic and biofilms [1–6]. This is the first study presenting an opaque cell-specific proteome dataset. Opaque form of *C. albicans* is still one of the least studied morphological forms of *C. albicans* [7]. Not many studies are available on the pathogenicity, metabolic preferences, mating abilities, interactions with the host’s innate immune system, and sensitivity to environmental cues of opaque cells [7, 8]. Thus, it is an attempt to understand the morphophysiology of opaque form and its significance, especially in virulence and immune evasion is crucial. These results provide important insights into understanding the morphophysiological modulations in *C. albicans* (ATCC10231) opaque form growth using synthetic complete dextrose (SCD) medium. Quantitative proteomics for opaque form growth is included in our final article [6]. It would be helpful for the scientific community to investigate the regulation of phenotypic switching in *C. albicans*. It will also aid in studying how *C. albicans* evade the immune system.

### Data description

This is the raw data from our published study on the changes in morphophysiology and molecular architecture under opaque form of *C. albicans* (ATCC10231) [6]. The SWATH-MS (Sequential Window Acquisition of all Theoretical fragment ion spectra Mass Spectrometry) method creates a spectral library [9–12]. Information-dependent acquisition (IDA) files were obtained from combined peptide data of both forms of growth (opaque and white) and were used to create the spectral library. Further, the spectral library was used to get a list of differentially expressed proteins among test (opaque) and control (white) growth through SWATH acquisition. The details of the datasets linked to this article are given in Table 1. The dataset includes an expression analysis of all proteins under both the forms of growth. In addition to this, the functional annotation was carried out using CGD (*Candida* Genome Database), SGD (*Saccharomyces* Genome Database), David software, and UniProt Databases [6]. A detailed procedure for sample preparation is given in our article [6].

**Table 1:** Overview of data set related to the proteomic dataset of opaque form growth of *Candida albicans*

Label	Name of data file/data set	File types (file extension)	Data repository and identifier (DOI or accession number)
Data set 1	Proteomic data of opaque form responsive proteins of *Candida albicans* ATCC10231	Raw files (wiff and scan) and Peak files (mzML).	MassIVE (10.25345/C5BC3T662) [13]

### Limitations

- Current data is of in vitro growth of *C. albican*s opaque form growth.
- The micro-LC-MS platform used to generate the data has a lower resolution than nano-LC-MS/MS data or other high-resolution platforms.

## Data Availability

Mass spectrometry proteomic data were submitted to the MassIVE partner repository of the ProteomeXchange project and given the dataset accession number MSV000090980 [13] (10.25345/C5BC3T662).
